# Sensory nerve-secreted factors regulate basal keratinocyte function *in vitro*

**DOI:** 10.1093/iob/obaf009

**Published:** 2025-03-03

**Authors:** A Srivastava, A Noble, S L Payne

**Affiliations:** Department of Biomedical Sciences, Ontario Veterinary College, University of Guelph, ON N1G2W1, Guelph, Ontario, Canada; Department of Biomedical Sciences, Ontario Veterinary College, University of Guelph, ON N1G2W1, Guelph, Ontario, Canada; Department of Biomedical Sciences, Ontario Veterinary College, University of Guelph, ON N1G2W1, Guelph, Ontario, Canada

## Abstract

Basal keratinocytes in the skin epidermis respond to microenvironmental signals during homeostatic maintenance of the skin and following injury by proliferating, migrating, and differentiating to restore the epidermal barrier. Injuries to the skin can result in non-healing wounds, characterized by prolonged inflammation, failure to close, and chronic pain. The skin is densely innervated by peripheral sensory nerves, which contribute to the wound repair response. Although it is known that nerves are important for successful wound healing, the underlying cellular mechanisms of this phenomenon, and particularly the role of nerves in directing keratinocyte re-epithelialization, are poorly understood. To explore the relationship between sensory nerves and keratinocyte function *in vitro*, we cultured keratinocytes with conditioned media collected from dorsal root ganglia (DRG) in both homeostatic and post-wounding conditions and found that keratinocyte migration, proliferation, and phenotype, functions essential for re-epithelialization, were modulated by DRG conditioned media. Using a proteomic approach, we characterized the secretome of cultured DRG and identified key factors essential for wound healing, including extracellular matrix proteins, growth factors, and metabolic factors involved with ATP production, which was correlated with alternations in keratinocyte metabolism when cultured in DRG conditioned medium. Our results advance our understanding of the microenvironmental cues that direct keratinocyte function during normal cellular turnover and cutaneous wound healing *in vitro*, helping to drive the development of therapeutics that target dysregulated re-epithelialization.

## Introduction

The skin serves as the first line of defense for the body against external pathogens and stimuli, necessitating a rapid and robust response to injury (Kim and Dao 2023; Bain et al. 2024). Cutaneous wound healing consists of a series of complex physiological processes resulting in repair of the epithelial barrier function to restore skin homeostasis (Amadeu et al. 2004; Baum and Arpey 2006; Carlos et al. 2015; Wilkinson and Hardman 2020; Kim and Dao 2023; Bain et al. 2024). In the epidermis, keratinocytes are arranged in stratified layers with distinct phenotypes and molecular profiles (Sun et al. 1983; Johansen 2017; Jiang et al. 2020). The deepest layer of the epidermis, the *stratum basale*, contains cells, which are maintained in a mitotically active state (Roshan et al. 2015; Damen et al. 2021). To maintain epithelial homeostasis, basal keratinocytes periodically differentiate as they detach from the basement membrane and migrate vertically through the epidermal layers to terminate in the *stratum corneum* and slough off as dead stratified squamous cells (Jensen et al. 1999; Bikle et al. 2012; Damen et al. 2021). Following injury, wound healing requires re-epithelialization, which is characterized by spatiotemporally controlled migration, proliferation, and differentiation of basal keratinocytes to restore the damaged epidermal layer (Mirastschijski et al. 2004). Following a transient period of inflammation, keratinocytes migrate along newly formed granulation tissue, depositing a layer of proliferating cells to join the opposing sides of the wounds (Grinnell 1992; Baum and Arpey 2006). Aberrant keratinocyte activity at any stage of the wound healing process can result in a non-healing wound characterized by chronic inflammation and delayed or incomplete wound closure (Usui et al. 2008; Colenci and Patricia 2018; Wang et al. 2023; Bain et al. 2024). Non-healing wounds can cause chronic pain, infection, and a decrease in quality of life, posing a significant socio-economic burden for wound care (Scar Treatment Market Size, Share and Growth Report, 2030 2023). Dysregulated wound healing can also predispose individuals to various skin disorders such as psoriasis (Zhou et al. 2022) or hypertrophic and keloid scars (Usui et al. 2008).

The post-injury microenvironment contains a milieux of factors that influence the response to skin damage, and it is increasingly recognized that peripheral nerves play an important role in this response (Bonilla et al. 2002; Emmerson 2017; Cairns et al. 2018). The skin is richly innervated; containing sympathetic autonomic fibers in the dermis, and primary afferent sensory nerve fibers in the epidermis and subcutaneous tissues (Hilliges et al. 1995; Emmerson 2017; Talagas et al. 2020). Within the stratified epithelium are free nerve endings with cell bodies located within dorsal root ganglia (DRG) that are involved in signal transduction to discriminate between noxious and innocuous stimuli (Hilliges et al. 1995; Talagas et al. 2020). Keratinocytes form close contacts with sensory nerve endings, expressing pre-synaptic proteins, found in clear-core presynaptic vesicles, which can activate sensory neurons via SNARE-dependent vesicle release (Talagas et al. 2020). Patients that suffer from diseases or disorders associated with reduced innervation to the skin, such as diabetic neuropathy, are far more likely to develop ulcers or have wounds that fail to re-epithelialize, thus resulting in non-healing wounds (Engin et al. 1996; Richards et al. 1999; Smith and Liu 2002). Experimental removal or damage to peripheral nerves in animal models also delays re-epithelialization and therefore wound healing (Richards et al. 1999; Smith and Liu 2002). Individual cytokines and chemokines secreted by peripheral nerves have been identified to stimulate wound healing *in vitro* such as substance P (SP) and calcitonin gene-related peptide (CGRP), both of which accelerated epithelial cell migration (Blais et al. 2014). However, characterization of the full sensory nerve secretome and how these factors may play a role in wound healing has not yet been elucidated. Furthermore, despite compelling evidence for the dependency of cutaneous wound healing on innervation, little is known about the direct effect of sensory nerves on keratinocyte function.

Using sensory nerves derived from mouse DRG, we demonstrate that nerve-secreted trophic factors regulate keratinocyte proliferation, migration, and metabolism *in vitro* and that this regulation differs depending on keratinocyte state. Furthermore, we characterized the DRG secretome and identified a suite of extracellular matrix proteins and growth factors implicated in wound healing processes, as well as key regulators in canonical metabolic pathways involved in glycolysis and oxidative phosphorylation. Our results show that sensory nerves can directly regulate keratinocyte functions through the secretion of trophic factors, which may be leveraged to identify therapeutic targets to improve wound healing in non-healing pathologies.

## Materials and methods

### DRG harvest and culture 

Experimental procedures were performed following principles sponsored by the Canadian Council on Animal Care and approved by the University of Guelph Institutional Animal Care and Use Committee (Animal Use Protocol #4746). Female C57BL/6 mice (7–8 weeks old) were purchased from Charles River Laboratories and housed in cages with paper bedding on a 12-h light–dark cycle. Each cage housed at least two mice, and mice had *ad libitum* access to water and standard laboratory food. Before harvesting, glass coverslips were placed in the wells of either a 6-well plate (#1.5, 30 mm, Bioptechs, 10199-874) or 48-well plate (8 mm; Electron Microscopy Sciences, 50949314) and coated with 20 µg/mL poly-D-Lysine (PDL; Sigma–Aldrich, P6407-5 mg) for 2 h at 37°C. The PDL was then removed, followed by three 5-min rinses with phosphate-buffered saline (PBS; Gibco, SH30254FS). Laminin at a concentration of 10 µg/mL (Corning, CB-40232-1 mg) was added to each well, in the same volume as PDL, and incubated overnight at 37°C or 4°C for up to 14 days.

Mice were anesthetized in 5% isoflurane and euthanized using CO_2_. Animals were decapitated to expose the spinal column, which was removed and rinsed in chilled PBS. Excess muscle was removed using a number 4 scalpel (Excelta 181 SE, Feather #10 blade. 08-916-5A). Identical lengthwise incisions were made lateral to the spine midline on the ventral surface, allowing a small strip of tissue to be removed to expose the spinal cord. The spine was then bisected on the mid-sagittal plane, the spinal cord removed, and DRG collected using fine forceps (Fisherbrand, PL22 16100121) and placed in chilled PBS. Excess myelin was excised using a #3 scalpel (Fine Science Tools, 1003-12, No. 15 blade 08-916-5D) while viewed with an Olympus SZ-6145TR trinocular stereo microscope. DRG were further cleaned using a digestion cocktail of 1 mL of dispase II (Sigma–Aldrich, SCM133) and 0.5 mL 1X TrypLE (Gibco, 12605028) for 30 min at 37°C and 5% CO_2_.

During DRG digestion, the pre-coated plates were prepared for seeding by removing the laminin from all wells followed by three 5-min rinses in sterile PBS. Working in a sterile environment, each DRG was then seeded using fine forceps. Every DRG had 20 µL of complete neurobasal media (Neurobasal A medium (Gibco, 21103049), 2% (v/v) W-21 (Wisent, 003-015-XL), 1% (v/v) GlutaMAX (Gibco, c35050061), 1% (v/v) Pen/Strep (Wisent, 450-201-EL), 1.25 ng/mL nerve growth factor [NGF; Peprotech, 450-01-250 µg)] added and was left for 16–24 h at 37°C and 5% CO_2_ to allow for DRG adherence to the plate. Media was then topped up to a total of 2 mL (6-well plate), or 0.5 mL (48-well plate) and half-media changes were performed every second day to replenish nutrients and remove debris.

### Human epidermal keratinocyte culture

Human epidermal keratinocytes (HaCaT) were purchased from AddexBio (#T0020001) and cultured in Dulbecco's modified Eagles medium [DMEM F/12, (Wisent, 319-075-CL), 10% (v/v) fetal bovine serum (FBS; Gibco, 12483–022), 1% (v/v) Pen/Strep (Wisent, 450–201-EL)]. HaCaT cells from passages 15–20 were used in all experiments. Cells were kept at 37°C and 5% CO_2_ with full media changes performed every 2 days with 10% DMEM until the cells reached 70–90% confluency.

### DRG media collection

Lumbar DRG were seeded in either a coated 6-well or 48-well plate and cultured for 7 days to adhere and sprout neurites in complete neurobasal medium. One day before collection start, media was removed from each well and rinsed with pre-warmed PBS before albumin-free neurobasal was added [Neurobasal no phenol; Gibco, 12348017, 1% (v/v) GlutaMAX (Gibco, c35050061), 1% (v/v) Pen/Strep (Wisent, 450-201-EL), 1.25 ng/mL NGF (Peprotech, 450-01-250 µg)]. For sensory nerve-conditioned media (SN-CM) collection, 1 mL (6-well plate) or 0.25 mL (48-well plate) of spent media was removed and placed in a conical tube, snap-frozen in liquid nitrogen and stored at −80°C until use. Control media was unconditioned neurobasal collected at the same time points from coated wells that did not contain DRG. Prior to all analyses, the media was thawed on ice, pooled based on collection date and sample, filter sterilized (0.22 µm, Froggabio SF0-22PES) and centrifuged at 1000 ×*g* for 1 min at room temperature. A Bradford assay (BioRad, 5000112) was conducted per the manufacturer's protocol to ensure a minimum protein concentration of 2 µg/mL for all subsequent analyses.

### Characterization of the DRG secretome

#### Liquid chromatography-tandem mass spectrometry (LC-MS/MS)

Previously collected and clarified pooled SN-CM samples were sent to SPARC BioCenter, the Hospital for Sick Children, Toronto, Canada, for LC-MS/MS analysis. Protein identifications were accepted if they could be established at greater than 95.0% probability and contained at least three identified peptides. Protein probabilities were assigned by the Protein Prophet algorithm (Nesvizhskii et al. 2003). Proteins that contained similar peptides and could not be differentiated based on MS/MS analysis alone were grouped to satisfy the principles of parsimony (Käll et al. 2008). Bruker timsTOF Pro and the Evosep One LC system 30 samples per day method were used for both MS and LC, respectively, for data independent acquisition (DIA). All DIA document files were searched against *mus musculus* spectral libraries generated using UniProt proteome (Proteome ID: UP000000589, reviewed, Prosit (https://www.proteomicsdb.org/prosit/). The search parameters were as follows: maximum missed cleavage sites, 3; fixed modification, carbamidomethylation. Variable modifications include oxidation and acetylation. All DIA data were analyzed using Spectronaut 18.6 Direct DIA + software (Biognosys, Switzerland). Data were displayed as the log_2_ transformed protein abundance per sample. The final application of secretome analyses was conducted on the dataset obtained by removing proteins upregulated within the control sample of unconditioned media when compared to conditioned media, as these were identified as non-secreted factors. Additionally, protein quantity of similarly expressed proteins between the control and sensory nerve conditioned media were removed from the sensory nerve conditioned media secretome as background components of the culture media.

#### Cytokine analysis

One milliliter aliquots were taken from the pooled clarified samples and analyzed using a commercially available Mouse MMP 5-Plex Discovery Assay® (MilliporeSigma, Burlington, MA, USA) cytokine panel (Eve Technologies Corp, Calgary, AB, Canada). The 5-plex consisted of MMP-2, MMP-3, MMP-8, proMMP-9, and MMP-12. Assay sensitivities of these markers range from 1.6 to 8.4 pg/mL. Base complete neurobasal was used as a control. The multiplexing analyses were performed using the Luminex™ 200 system (Luminex, Austin, TX, USA) by Eve Technologies Corp. Cytokine quantity was plotted as a fold change relative to the control. Samples with proteins out of the detection range without specification of being above or below were assigned a value of zero. Out of range (too low to be detected), analytes were assigned the value of the lowest standard. Unpaired *t*-tests were conducted between the control and SN-CM per observed analyte.

#### Gene ontology and network analyses

Proteins expressed in the lumbar DRG secretome were analyzed using the protein analysis through evolutionary relationships classification system (PANTHER; https://pantherdb.org/; version 18.0). This system classified identified proteins based on three main ontological terms: biological processes, molecular functions, and cellular component using phylogenetics (Thomas et al. 2021). To display protein interactions, proteins were uploaded into STRING database (https://imagej.net/software/fiji/; version 12.0) with confidence scores set to a minimum of 0.7 for high-confidence interactions.

### Scratch assay

The methodology used in this study was adapted from Liang et al. (2007). Briefly, HaCaT cells were seeded at 3.0 × 10^5^ cells per well in a 6-well plate in 10% DMEM F/12 and grown for 1–2 days to form a 90–95% confluent monolayer. Cell morphology and monolayer uniformity were assessed prior to wounding. To facilitate consistent imaging of the same site over multiple time points, a cross was etched on the back of the plate to identify the region of interest. A vertical scratch was manually created along the center of each well using a P1000 pipette tip, using the etched cross as a guide. Media was then removed, and cells were washed with pre-warmed PBS. The PBS was removed and conditioned media along with serum reduced media in a 1:1 ratio [DMEM F/12, (Wisent, 319–075-CL), 5% (v/v) FBS (Gibco, 12483–022), 1% (v/v) Pen/Strep (Wisent, 450-201-EL)] was added to the cells. This ratio was chosen as it maintains keratinocyte viability in culture for up to 7 days (Figure S1). The scratch site was imaged at the same location at time 0, 24, 48, and 72 h after wounding using the EVOS XL Core Imaging system (AMEX1000) at 4x magnification. The scratch area, wound coverage of total area and standard deviation (SD) of the scratch width were quantified using an ImageJ (Rasband, W.S., ImageJ, U.S. National Institutes of Health, Bethesda, MD, USA, http://imagej.nih.gov/ij/) plugin adapted from Suarez-Arnedo et al. (2020). The rate of cell migration and percentage of wound closure were calculated according to (equation 1) and (equation 2), respectively, based on calculations from a protocol by Grada et al. (2017).


(1) \begin{eqnarray*}
\left( {\mathrm{a}} \right){\mathrm{Rate\,\,of\,\,\mathrm{migration}\,\,}} = \frac{{Wi\,\, - \,\,Wt}}{{t\Delta }},
\end{eqnarray*}

(2) \begin{eqnarray*}
({\mathrm{b}})\,{\mathrm{Wound}}\,{\mathrm{closure}}\,{\mathrm{\% \,\,}}\left( {\frac{{At = 0\,\, - \,\,At = \Delta }}{{At = 0}}} \right) \times 100,
\end{eqnarray*}

where W*i* is the initial wound width, W*t* is the wound width at *n* h both in μm, and *t* is the time span of the assay at *n* in h. *At* = 0 is the initial wound area, *At = Δt* is the wound area after *n* h of the initial scratch, both in μm^2^.

### Immunocytochemistry and staining

At either 24, 48, or 72 h of culture in conditioned media, keratinocytes were fixed with 4% paraformaldehyde solution for 20 min followed by PBS washes. To perform ICC, cells were treated with 1 mL (6-well) or 0.2 mL (48-well) per well of blocking solution [0.1% v/v Triton-X-100 (VWR, 97063–864) and 5% w/v (mg/µL) bovine serum albumin (BSA; Wisent, 800–095-EG) in PBS] for 1 h at room temperature. Blocking solution was removed and replaced with primary antibodies diluted in blocking solution and incubated overnight at 4°C. After 16–24 h, the primary antibody solution was removed; cells were rinsed three times for 5 min each with PBS and then treated with secondary antibodies diluted in blocking solution for 2 h at room temperature protected from light. The secondary solution was removed, cells were rinsed three times for 5 min before adding a 4′,6-diaminido-2-phenylindole (DAPI) solution (1:100; Invitrogen, D1306) for 10 min at room temperature in a darkened incubator chamber. The DAPI solution was removed, the cells were washed twice for 5 min and left in 0.5 mL (6-well) or 0.2 mL (48 well) of PBS for imaging.

Primary antibodies used along with their dilutions are as follows; rat anti-Ki67 (1:250; Invitrogen, 14-5698-82), mouse anti-cytokeratin 14 (1:250, Invitrogen, LL002) and rabbit anti-cytokeratin 10 (1:250, ThermoFisher, MA5-32183). Secondary antibodies used along with their dilutions are as follows: AlexaFluor488 goat anti-rat (1:500; ThermoFisher, A11006), AlexaFluor488 goat anti-rabbit (1:500; ThermoFisher, A11008), and AlexFluor546 goat anti-mouse (1:500; ThermoFisher, A11003).

To measure mitochondrial activity in post-scratch keratinocytes in the presence of SN-CM, at 24, 48, and 72 h, post-scratching cells were incubated with 200 nM MitoTracker Green FM (ThermoFisher, M7514) for 45 min at room temperature in a chamber concealed from light. The MitoTracker solution was then removed; cells were washed twice for 5 min with PBS, and left in PBS for imaging.

### Image analysis

Cells were imaged using a confocal microscope (Olympus FV1200) at 10x magnification or a fluorescent microscope (ThermoFisher EVOS M7000) at 10x. Three images per well were taken if in a 6-well plate and two images per well were taken if in a 48-well plate unless otherwise stated. Images were analyzed and quantified using ImageJ software. For Ki67 quantification, a threshold was set for images taken from each channel to identify individual cell nuclei. Using the *Analyze Particles* function, nuclei were counted in the DAPI channel. Any nuclei not included were manually accounted for using the *Cell Counter* function. DAPI and Ki67 signals were then merged, and co-staining nuclei were manually counted using the *Cell Counter* function. The percentage of positive Ki67 nuclei was determined by dividing the total number of cells by the amount of co-stained Ki67 positive and DAPI cells. For K14/K10 and MitoTracker quantification, images were first converted to 8-bit format. A threshold value was set at each timepoint for both channels separately and the *Create Selection* function was used to select positive pixels for each channel, followed by the *Measure* function to quantify the total number of positive pixels. This approach to protein expression quantification has been previously used by others (Inman et al. 2005; Jin et al. 2020). A width of 1300 µm to either side of the wound area was selected for quantification. This process was repeated for each channel per image. Final quantification was presented as total % positive pixels normalized to cell count.

### Seahorse XF Real-time ATP Rate assay

Bioenergetic profiling of keratinocytes with and without culture in SN-CM was conducted using the Agilent Seahorse XFe24 analyzer with the ATP rate assay protocol outlined by the manufacturer with some adjustments (Agilent Technologies, Santa Clara, CA) to measure oxygen consumption rates and extracellular acidification and obtain total ATP production in homeostatic keratinocytes. Respiratory parameters were corrected for non-mitochondrial respiration and background signaling from the instrument using 4.3 µM Rotenone/Antimycin A as well as 150 µM of Oligomycin, where both reagents were part of the commercially available Seahorse XF Real-Time ATP Rate Assay (Agilent, 103592 100). Buffered assay medium was composed of 40 mL Seahorse XF DMEM Medium (Agilent, 103575–100, 10 nM (v/v) XF glucose (Agilent, 103577–100), 1 nM (v/v) pyruvate (Wisent, 600-110-EL), 2 mM (v/v) L-glutamine (Gibco, 25030–081)). Parameters included the total ATP production (ATP index) through measurement of glycolytic and mitochondrial pathways. Experiments were conducted at 37°C and included an input buffer factor of 2.3 at a pH of 7.4 ± 0.20. The report was generated using Wave 2.6.3 and report generator for ATP rate assay provided by Agilent Technologies. Data were normalized to cell count with a scale factor of 10,000.

### Statistical analysis

Both statistical analysis and graphing were performed in Prism GraphPad software (https://www.graphpad.com). Quantification of scratch assay results were analyzed using a mixed-design analysis of variance (mixed-ANOVA) using multiple comparisons between two groups at four separate timepoints. Proliferation, keratin expression, and MitoTracker data were analyzed using an unpaired Student's *t*-test per timepoint. One-way ANOVA analysis was used for Seahorse XFe24 data with multiple comparisons to compare the differences between metabolism as well as total ATP production. One outlier was removed from the Seahorse XF analysis from Sample A as a Grubbs outlier test determined it had a *z*-score of 1.713 and was considered a significant outlier (*P* < 0.05). Cytokine array data were presented as fold change relative to control and was analyzed using an unpaired t-test per selected cytokine. Data are shown as mean ± SD unless otherwise noted. Statistical significance was set at *P* < 0.05.

## Results

### Sensory nerve conditioned medium significantly alters keratinocyte migration and phenotype after mechanical scratch

**Fig. 1.**
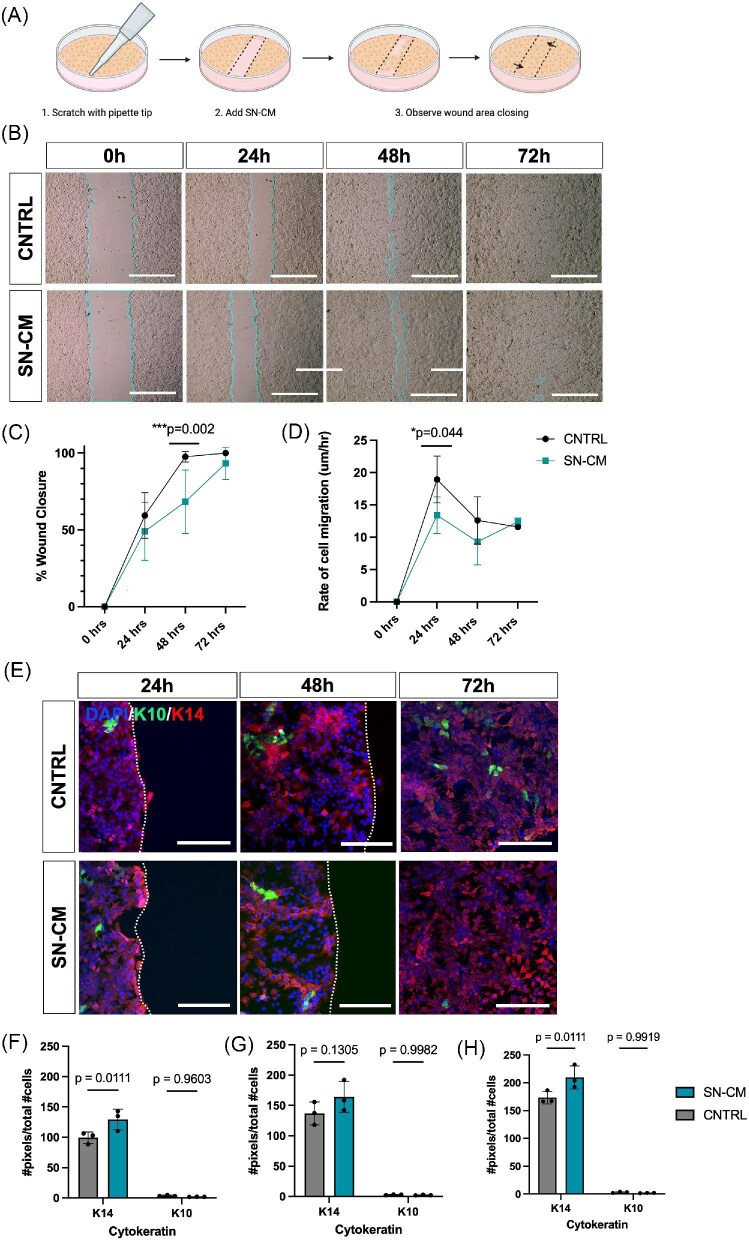
Sensory nerve conditioned media delays wound closure and decreases keratinocyte migration without affecting K10 expression. **(A)** A schematic representation of the scratch assay. **(B)** Representative brightfield images of keratinocytes from the SN-CM and control (CNTRL) group at 24, 48, and 72 h after injury. Scale bar = 800 µm. Wound margins are indicated by the teal line. **(C)** Percent wound closure over 72 h. Culture with SN-CM significantly decreased wound closure at 48 h compared to control (***P = 0.0018, *n* = 4 biological replicates). **(D)** Rate of keratinocyte migration over 72 h. Culture with SN-CM significantly reduced the rate of migration at 24 h (**P = 0.0442, *n* = 4 biological replicates). (**E**) Representative images of keratinocytes after scratch immunostained for K14 (red) and K10 (green). Wound margins are indicated by the white line. Scale bar = 150µm. Quantification of K14 and K10 expression at (**F**) 24, (**G**) 48, and (**H**) 72 h after injury (*n* = 3 biological replicates). Values are expressed as the mean ± SD.

Previous work has suggested that sensory nerves secrete trophic factors to promote wound healing (Engin 1998; Bonilla et al. 2002; Saygili et al. 2010; Blais et al. 2014; Cairns et al. 2018; Talagas et al. 2020). To determine the effect of the nerve secretome on keratinocyte function, basal keratinocytes were cultured in SN-CM from mouse DRG and keratinocyte migration, proliferation, and differentiation, key events of re-epithelialization, was assessed *in vitro*. Mouse and human DRG have a conserved transcriptomic profile, including transcription factors, G-protein coupled receptors, and ion channels and thus the mouse DRG represent a good model for human sensory nerves (Chiu et al. 2014). To assess keratinocyte migration, an *in vitro* wound-healing assay was used to measure wound closure and cell migration speed after scratch injury and culture in SN-CM for 24, 48, and 72 h (Fig. 1A and B). To ensure that any changes in wound closure, we observed, were due to changes in keratinocyte migration and not proliferation, cells were grown in serum-reduced medium and we confirmed that proliferation was minimal (<5% of cells) in both the SN-CM and control group using immunocytochemistry (Figure S2). At 24 h, the % wound closure was similar in the control and SN-CM group; however at 48 h, we observed a significant decrease in wound closure in keratinocytes cultured in SN-CM compared to the control, suggesting that SN-CM slows wound closure at this timepoint (Fig. 1C). Regardless of culture condition, near closure was achieved by 72 h post-wounding (Fig. 1C) although while complete wound closure of all scratches in control conditions was seen by 72 h, those cultured in SN-CM failed to close 55.6% of the time. We next analyzed the rate of keratinocyte migration by determining the change in wound area and dividing it by the time in hours (equation 1), finding that at 24 h, the rate of keratinocyte migration when cultured in SN-CM was significantly slower (12.1 µm/h, *P* = 0.0442) when compared to the control group (19.5 µm/ h). Similar to the change in % wound area, this difference in cell speed resolves itself at 72 h as the wound nears complete closure (control speed of 11.58 µm/h and SN-CM speed of 12.4 µm/h) (Fig. 1D). These results suggest that trophic factors contained in SN-CM affect keratinocyte migration by reducing keratinocyte speed, thus delaying overall scratch wound closure *in vitro*.

During homeostasis, keratinocytes maintain the epidermal barrier and express epithelial keratins, forming a network of intermediate filaments to support keratinocyte activity (Sun et al. 1983). Keratins exhibit a tightly regulated spatial expression pattern within the epidermis, which is maintained by the differentiation program within the skin (Byrne et al. 1994; Alam et al. 2011). Keratinocytes within the proliferative *stratum basale* express keratin 14 (K14), whereas keratinocytes in the postmitotic suprabasal layers express keratin 10 (K10) to signify initiation of the differentiation stage (Moll et al. 1982; Byrne et al. 1994; Alam et al. 2011). Keratinocyte differentiation during wound healing is also an integral part of re-epithelialization through the restoration of a functional stratified epidermis, providing a pool of suprabasal cells to close over the wound surface as the cells migrate (Bikle et al. 2012; Zhang et al. 2019). We quantified expression of K14 and K10 in keratinocytes at the wound edge after scratch to determine if the change in keratinocyte migration was accompanied by changes in the K14+ basal keratinocyte phenotype to a suprabasal layer phenotype (K10+). Overall, we observed that most cells, as expected, express K14 with minimal K10 expressing-cells in the population (Fig. 1E). Interestingly, we found that at 24- and 72-h post-scratch there was a statistically significant increase in K14 + cells in the SN-CM group compared to the control but no difference in the number of K10+ cells at any timepoint (Fig. 1E–H). These data show that culture of scratch wounded keratinocytes in SN-CM decreases keratinocyte migration and increases expression of the basal keratinocyte marker K14.

### Sensory nerve-conditioned media decreases keratinocyte proliferation in homeostatic conditions

**Fig. 2.**
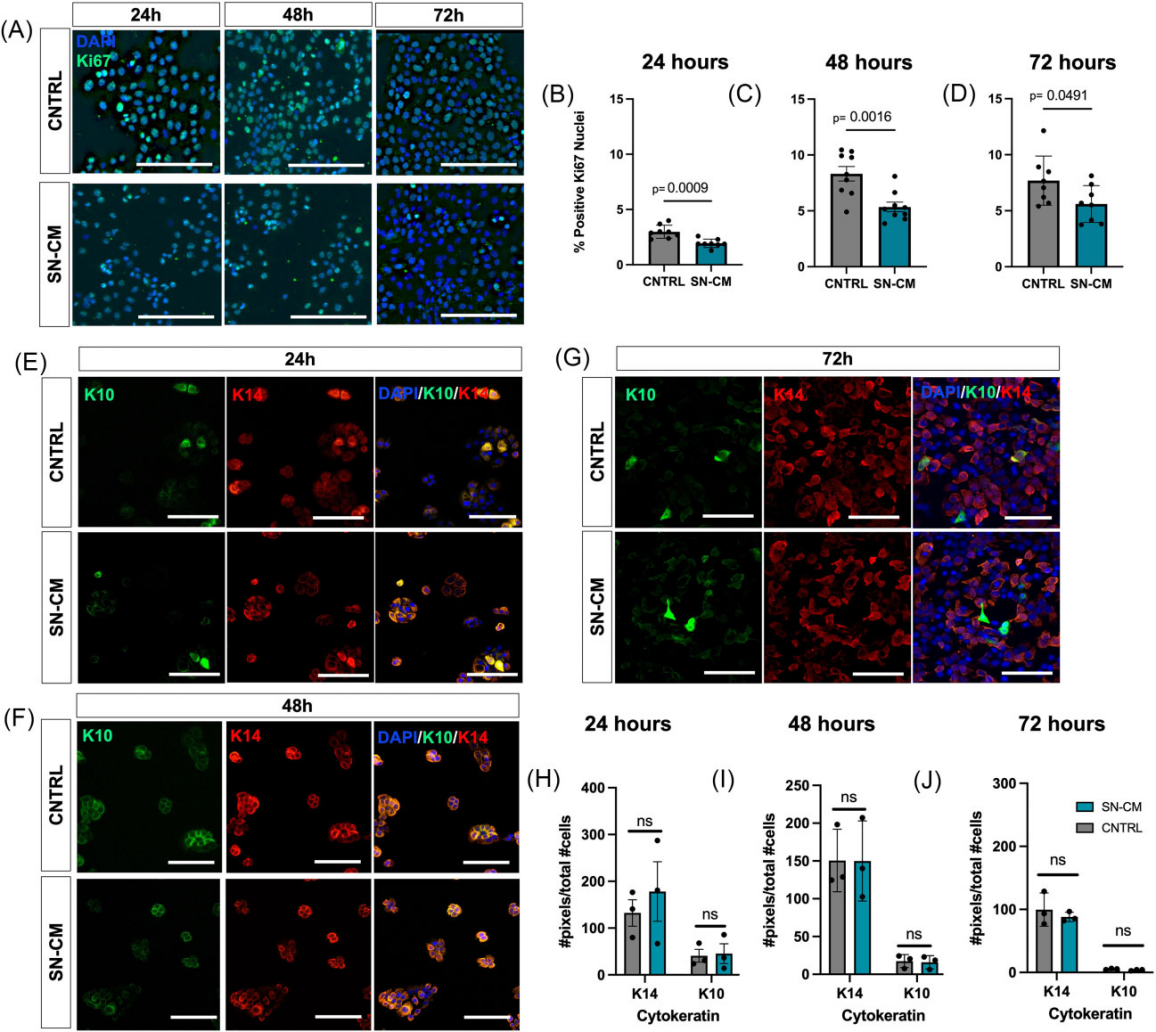
Culture in sensory nerve conditioned media reduces homeostatic keratinocyte proliferation. **(A)** Representative fluorescent images of keratinocytes cultured in SN-CM and control media at 24, 48, and 72 h of culture (blue = DAPI, green = Ki67). Scale bar = 100 µm. Quantification of keratinocyte proliferation in culture with SN-CM at **(B)** 24 (*P* = 0.0009, *n* = 8), **(C)** 48 (*P* = 0.0016, *n* = 9), and **(D)** 72 (*P* = 0.0491, *n* = 8) h. Culture with SN-CM significantly decreased cell proliferation as measured by % Ki67 positive nuclei at all-time points. Representative fluorescent images of keratinocytes cultured in SN-CM and control media at (**E**) 24, (**F**) 48, and (**G**) 72 h of culture (blue = DAPI, green = K10, red = K14). Scale bar = 150 µm. Quantification of K14 and K10 expression at (H) 24, (I) 48, and (**J**) 72 h of culture in SN-CM or control medium (*n* = 3). Values are expressed as mean ± SD.

After determining that culture in SN-CM slows the migration of keratinocytes and increases K14 expression after scratch wounding, we next wanted to investigate whether SN-CM affects homeostatic, non-injured keratinocytes. Regulation of keratinocyte proliferation is important for both maintaining epithelial homeostasis and re-epithelialization after injury, as new cells are required to repopulate the damaged epidermal barrier. We cultured keratinocytes in SN-CM or control medium and quantified cell proliferation and differentiation after 24, 48, and 72 h of culture (Fig. 2A). We found that while overall keratinocyte proliferation increased over time in both groups, at all timepoints keratinocytes cultured in SN-CM had a significantly reduced percentage of positive Ki67 cells compared to the control (Fig. 2B–D). This result suggests that soluble factors contained in SN-CM can regulate keratinocyte proliferation *in vitro*. We also analyzed K14 and K10 expression in intact keratinocyte monolayers cultured in SN-CM or control medium for up to 72 h, observing that as expected the majority of cells expressed K14 with minimal K10 expression (Fig. 2E–G). Culture of keratinocytes in SN-CM produced no significant difference in keratin expression compared to keratinocytes cultured in control medium at all timepoints investigated (Fig. 2H–J). These results demonstrate that sensory nerve conditioned media decreases keratinocyte proliferation in the absence of a scratch wound but, unlike in post-scratch conditions, has no effect on keratin expression.

### Proteomic profiling of the mouse lumbar sensory nerve secretome

**Fig. 3.**
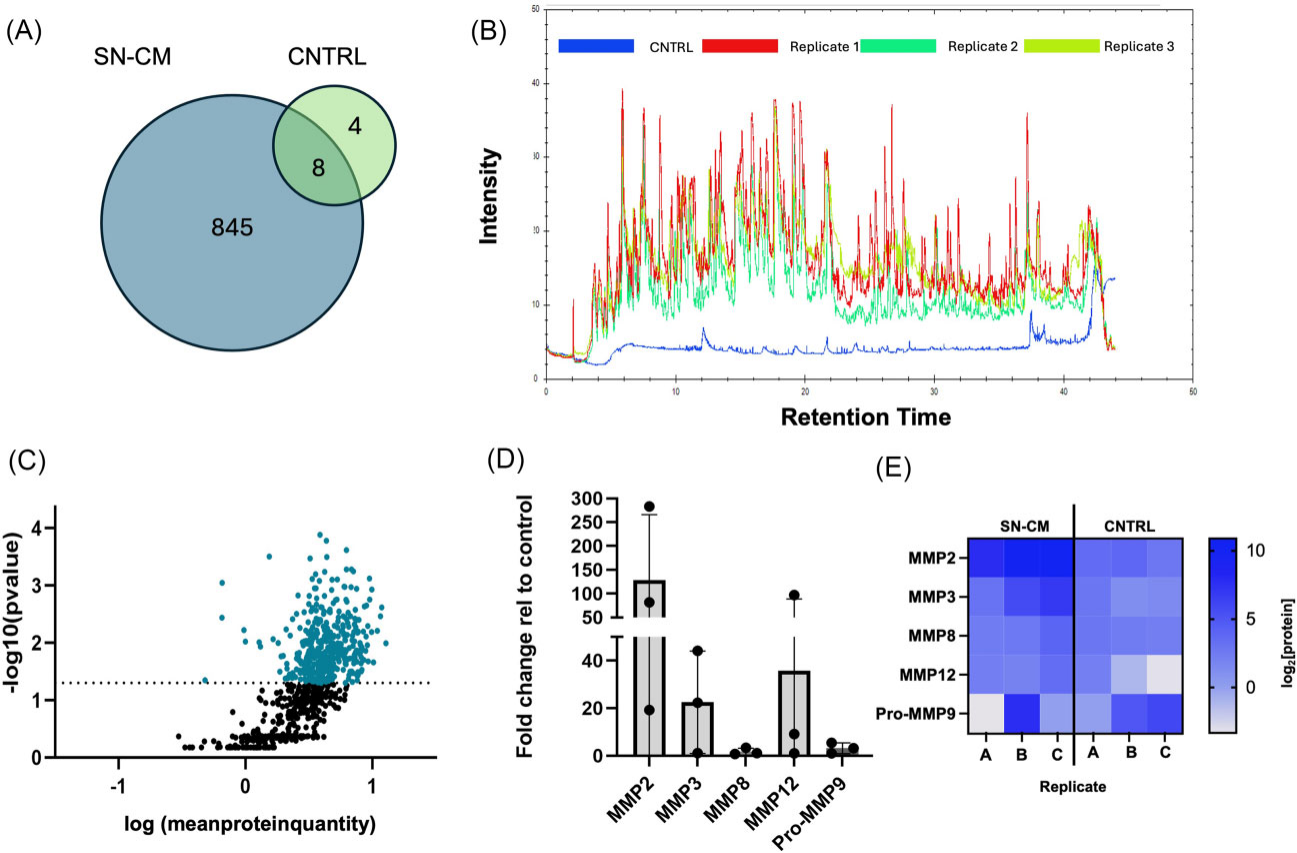
Proteomic characterization of the sensory nerve secretome identified trophic factors associated with wound healing. **(A)** Venn diagram of total number of proteins identified in SN-CM (845) compared to the control (Wilkinson and Hardman 2020) and shared proteins (Jiang et al. 2020). **(B)** TIC overlay plot sum of overall ion intensity (10^6^) during fragmentation by the total retention time across all three replicates of SN-CM and the control. Peaks represent protein detection, and intensity correlates to protein abundance. **(C)** Volcano plot of log-transformed proteomic data from SN-CM relative to the control (*n* = 3; *P* < 0.05). Dashed horizontal line represents the p-value cut-off, where all points above were considered significantly upregulated within SN-CM. **(D)** Cytokine array quantification of MMP concentration in SN-CM displayed as a fold change relative to the control of base unconditioned media (CNTRL) (*n* = 3 biological replicates). (**E**) Heat map of log transformed protein concentrations in SN-CM and CNTRL from the MMP panel.

After determining that SN-CM alters keratinocyte function in both post-wounding and homeostatic culture conditions, we next characterized the secretome of DRG to identify candidate trophic factors responsible for these effects. Previous work has suggested that peripheral nerves promote wound healing and tissue repair by secreting bioactive molecules such as SP (Blais et al. 2014), CGRP (Engin 1998), and NGF (Generini et al. 2004), which regulate inflammation, angiogenesis, and cellular proliferation (Generini et al. 2004; Gordon 2009). However, profiling of the DRG secretome has not been published, leaving a critical knowledge gap in identifying the specific molecular players involved in nerve-mediated wound healing. We collected SN-CM from DRG and used tandem mass spectrometry, which is widely used to identify unknown ions in untargeted proteomics to detect a large-scale quantity of proteins, thus allowing for the quantification of DRG secreted factors. DIA LC-MS/MS identified a total of 849 proteins across three biological replicates of SN-CM (Fig. 3A and B). Replicate 1 contained 801 of the 849 expressed proteins, replicate 2 contained 754 proteins, and replicate 3 contained 789 proteins (Figure S3). Overall, as expected, the control of base unconditioned media expressed low protein abundance compared to SN-CM (Fig. 3A and B, Figure S3). There was a total of 12 proteins detected within the control group. Four of these proteins were upregulated in the control and therefore removed from the data set of all samples. The total protein quantity of the 8 remaining proteins found in all sample replicates was subtracted from the SN-CM groups to determine overall protein quantity relative to proteins detected in base unconditioned media. Therefore, we concluded that DRG secrete 845 of the 849 identified proteins (Fig. 3A). The sum of the total ion current (TIC) across the retention time, which denotes the abundance of all ions within the spectrum in a certain sample, showed lower peaks and intensity in the control compared to experimental groups (Fig. 3B). In contrast, SN-CM replicates showed similar peaks and troughs throughout the retention time at a higher intensity, indicating higher protein abundance as well as a similarity of proteins expressed between the experimental samples (Fig. 3B). Of the total proteins detected, 442 proteins were significantly enriched within SN-CM compared to the control (Fig. 3C). Within our identified protein dataset, we noted the secretion of several factors previously reported to be involved in wound healing including regulators of the ECM such as collagen type I and IV (Abreu-Velez and Howard 2012), tissue inhibitor of MMPs (TIMP-2) (Vaalamo et al. 1999), fibrillin-1 (Isogai et al. 2003), bone morphogenic protein-1 (BMP-1) (Muir et al. 2016), fibroblast growth factor-1 (FGF-1) (Oladipupo et al. 2014) as well as cadherin-10 (Crawford and Dagnino 2018), which is involved in cell adhesion of both keratinocytes and dermal fibroblasts (Table 1).

**Table 1.** Significantly enriched proteins in SN-CM with known roles in skin wound healing

**Protein**	**Function**	**Mean protein abundance**
TIMP2	Promotes keratinocyte migration, regulates MMP activity (Generini et al. 2004)	3.41
Collagen type I	Scaffold for cell attachment (Zhang et al. 2019)	3.89
Collagen type IV	Formation of basement membrane following injury (Zhang et al. 2019)	1.61
Fibrillin-1	Regulation of matrix metabolism through modulation of TGF-β (Gordon 2009)	4.16
BMP-1	Processes collagens to regulate ECM formation (Abreu-Velez and Howard 2012)	5.52
Cadherin-10	Cell adhesion (Isogai et al. 2003)	1.91
FGFR-1	Angiogenesis following injury^113^	2.34
Serpina3n	Accelerates migration in epidermal cells (Rigoulet et al. 2020)	1.04
MMP2	Cell adhesion and collagen degradation (Mäkelä et al. 1999)	128.1
MMP3	Collagen degradation (Rao et al. 2006)	22.54
MMP8	Cleaves collagens I, III, IV (Demeter et al. 2022)	1.78
MMP12	Degradation of basement membrane (Laronha and Caldeira 2020)	35.72
Pro-MMP9	Precursor to MMP9 (Gordon 2009)	3.21

To confirm our findings from mass spectrometry and identify proteins with known roles in wound healing in the SN-CM that might be present at concentrations below the detection threshold of LC-MS/MS, baseline unconditioned media and SN-CM samples were analyzed using a 5-Plex MMP Discovery Assay (Eve Technologies) (Fig. 3D and E). Cytokine arrays use highly sensitive antibody probes to detect pre-determined molecules based on a selected panel (Skalnikova et al. 2017). Regulation of MMP activity is critical for re-epithelialization, particularly MMP2, which has been shown to be upregulated immediately following injury to promote keratinocyte migration and wound closure (Mäkelä et al. 1999; Xue and Jackson 2008; Stevens and Page-McCaw 2012). Keratinocytes themselves release MMP2, MMP9, and MMP13 following injury, largely in response to pro-inflammatory cytokines such as IL-1β and TNF-α, which activate intracellular signaling pathways including NF-κB and MAPK (Mohan et al. 2002; Hattori et al. 2009; Stevens and Page-McCaw 2012; Rastogi et al. 2013). Using the MMP panel, we detected proteins from the SN-CM in a concentration range of 4.04 pg/mL (MMP-8) to 1902 pg/mL (MMP-2). All 5 MMPs involved in cutaneous wound healing were identified in SN-CM (Fig. 3D). Although we detected an increase in fold change between the control and SN-CM groups, there was no statistically significant increase in concentration for any analyte, which may be due to variability between biological replicates of SN-CM (Fig. 3E). Overall, combining both the LC-MS/MS and cytokine array, we identified a suite of proteins secreted by DRG in culture, many of which have important roles in wound healing (Table 1).

**Fig. 4.**
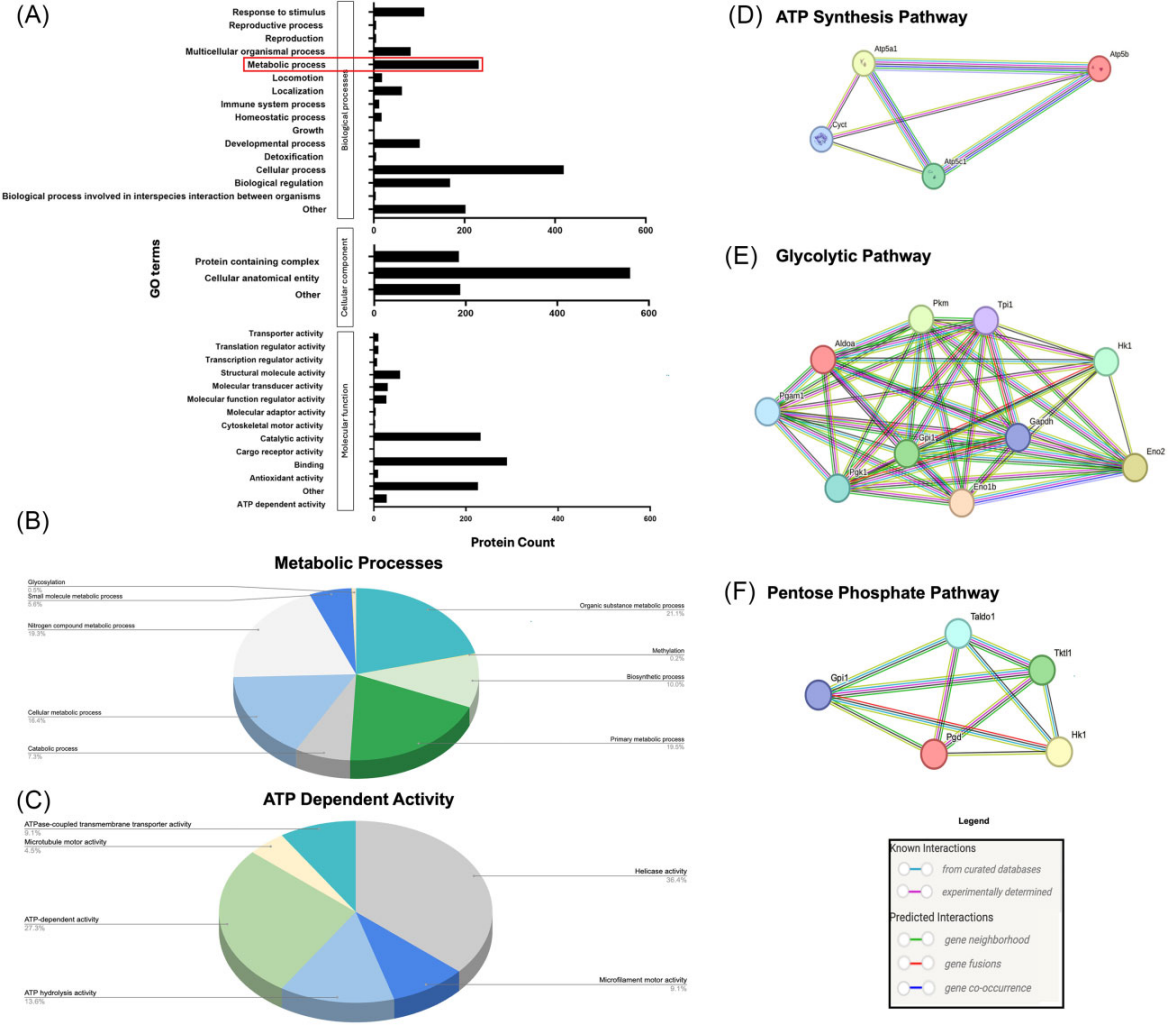
GO terms of proteins detected in sensory nerve conditioned media and subclassification of metabolic processes. **(A)** GO terms for biological processes, cellular components, and molecular function were identified using the PANTHER 18.0 classification system (*n* = 3). Subclassification of **(B)** metabolic processes and **(C)** ATP dependent activity from highlighted metabolic processes category identified using the PANTHER database. Protein–protein interactions of all detected SN-CM analytes within the **(D)** ATP synthesis pathway, **(E)** glycolytic pathway, and **(F)** pentose phosphate pathway identified using the STRING database. All colored nodes represent direct interactions.

To further link the identified proteins of the SN secretome to cellular functions involved in wound healing, proteins detected within SN-CM were mapped to gene ontology (GO) terms based on the functional annotation from the PANTHER classification system against the *mus musculus* genome (Thomas et al. 2021). This database is designed to classify proteins and their genes to identify protein properties, families, subfamilies, and functions. Within biological processes, top hits included cellular processes (419 proteins), biological regulation (168 proteins), and metabolic processes (231 proteins). Within the cellular components class, the GO term with the most matched proteins was cellular anatomical entity (559 proteins). Within molecular function, the top GO terms were catalytic activity (232 proteins), binding (289 proteins), and structural molecule activity (57 proteins) (Fig. 4A). Interestingly, PANTHER subclassification of biological processes identified subgroups of proteins involved in metabolic processes, which we parsed out further due to the known importance of metabolic regulation during re-epithelialization (Baum and Arpey 2006; Wilkinson and Hardman 2020; Kim and Dao 2023; Bain et al. 2024). Within metabolic processes, we identified 188 proteins involved in cellular metabolic processes, which include energy storage, conversion, and production pathways such as glycolysis, gluconeogenesis, and the pentose phosphate pathway (Fig. 4B). A further 115 of the identified proteins are involved in biosynthetic processes, which include ATP production molecules such as the alpha, beta, and gamma subunits of ATP synthase. Another subclassification of metabolic processes identified was ATP dependent activity, which categorized 22 of the proteins found in SN-CM that are involved in processes such as hydrolysis, microtubule motility, and ATP mediated helicase activity (Fig. 4C). Through further proteomic analysis of SN-CM using the STRING protein–protein association database (v.12), we were able to identify factors within SN-CM from core pathways involved in ATP production, such as cytochrome-c (Fig. 4D), pyruvate kinase (PKM) (Fig. 4E), and hexokinase-1 (Fig. 4E and F). These pathways are known to be involved in ATP production (Takahashi et al. 2013; Hamanaka and Chandel 2013; Plitzko and Loesgen 2018; Yan et al. 2024) and were amongst the significantly upregulated proteins detected in SN-CM by our LC-MS/MS. The STRING analysis also identified detected metabolic proteins that are directly or physically related to one another, indicating a strong association between the proteins found in SN-CM (Fig. 4D–F) (Takahashi et al. 2013).

### Sensory nerve-conditioned media alters keratinocyte metabolism in both post-wounding and homeostatic states

**Fig. 5.**
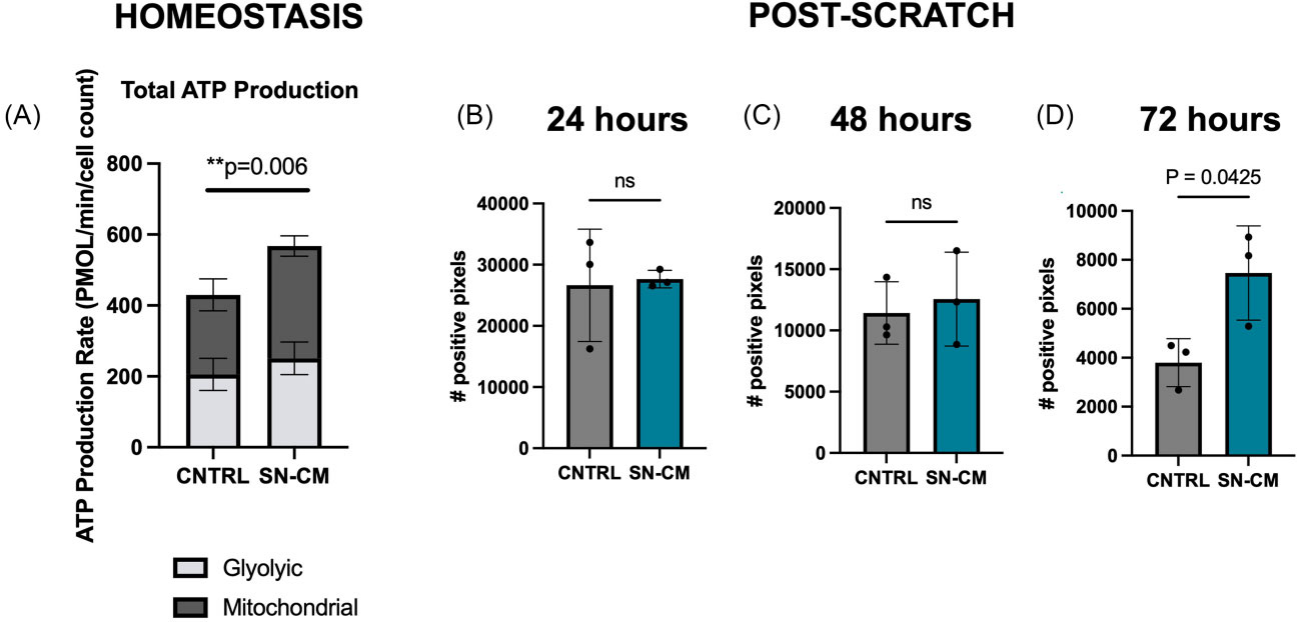
Sensory nerve conditioned medium increases total ATP production in homeostatic keratinocytes and mitochondrial activity after scratch injury. **(A)** Quantification of mitochondrial and glycolytic ATP production rates for real-time measurement of cellular ATP production rates and quantitative phenotype of cellular energy profile of keratinocytes. Analysis shows a significant increase (*P* = 0.006) in total ATP production between the control (*n* = 3) and keratinocytes cultured in SN-CM (*n* = 3). No significant differences were found when comparing the ATP source between groups. Biological replicates represent samples of SN-CM harvested from separate mice. Values are expressed as mean ± SD. Quantification of MitoTracker fluorescence at (B) 24, (C) 48, and (D) 72 h in keratinocytes cultured in CNTRL or SN-CM following scratch injury. At 72 h, post-scratch cells cultured in SN-CM had a significantly greater MitoTracker signal, indicating more mitochondrial activity. Values are expressed as mean ± SD, *n* = 3 biological replicates.

As our functional mapping of proteins identified a significant number of proteins involved in cell metabolism, we wanted to further explore this function as a potential means of keratinocyte regulation by sensory nerves. Keratinocyte metabolism plays a crucial role in supporting key energy extensive processes required for wound healing, including cell proliferation, migration, and differentiation (Hamanaka and Chandel 2013; Plitzko and Loesgen 2018; Yan et al. 2024). Energy generated through metabolic pathways fuels cytoskeletal remodeling, protein synthesis, and secretion of growth factors and cytokines essential for tissue repair (Yan et al. 2024). Additionally, given that culture of keratinocytes in SN-CM significantly reduces keratinocyte migration and proliferation, we wanted to confirm that these observations were not due to an overall negative effect of SN-CM on keratinocyte viability through the loss of mitochondrial function or ATP production. We characterized the bioenergetic profile of homeostatic keratinocytes in SN-CM after 72 h in culture with SN-CM or control media. This assay measures total ATP production in living cells and distinguishes ATP production from mitochondrial respiration or glycolysis by detecting oxygen consumption rates (OCRs) and glycolytic extra-cellular acidification rates (ECARs), respectively (Szklarczyk et al. 2023). Oxidative phosphorylation consumes oxygen, driving the OCR, and the conversion of glucose to lactate leads to the extrusion of a proton, captured by the ECAR (Szklarczyk et al. 2023). Our results show that the total ATP production rate of keratinocytes in SN-CM was significantly increased (*P* = 0.006) when compared to culture in control medium (Fig. 5A). Interestingly, there was no significant difference in the ratio of ATP produced from mitochondrial oxidative phosphorylation and glycolysis between the two groups, indicating that while culture in SN-CM increases total ATP, it does not alter the metabolic pathways responsible for ATP production (Takahashi et al. 2013). Due to culture plate compatibility issues with the Seahorse XFe24, we were unable to repeat this assay on keratinocytes after scratch injury; however as an alternative, we investigated mitochondrial activity using MitoTracker labeling of active mitochondria in live cells (Fig. 5B–D). Our results show that at 72 h post-scratch, keratinocytes cultured in SN-CM had a significantly higher MitoTracker signal than cells cultured in control media (Fig. 5D). Overall, from these experiments, we can conclude that the addition of SN-CM to cultured keratinocytes does not negatively affect keratinocyte metabolism or the ratio of ATP source (i.e., oxidative phosphorylation or glycolysis) and significantly increases total ATP production when keratinocytes are in a homeostatic state and mitochondrial activity post-scratch when wound closure is largely complete. This increase in keratinocyte metabolism may occur via the presence of key metabolic factors involved in ATP producing pathways, such as glycolysis, within the SN-CM.

## Discussion

### Sensory nerve secreted factors regulate keratinocyte function variably depending on cellular state

Spatiotemporal coordination of keratinocyte proliferation, migration, and differentiation is essential for both homeostatic maintenance of the skin and successful re-epithelialization, and disruption to the balance between these functions can result in non-healing wounds and other pathologies (Usui et al. 2008; Zhou et al. 2022). Previous work has shown that peripheral nerves are important for skin wound healing (Bonilla et al. 2002; Generini et al. 2004; Emmerson 2017; Cairns et al. 2018), although how nerves contribute to re-epithelialization, and the keratinocyte functions that support this process, is largely unknown. Others have demonstrated that the addition of isolated neuropeptides enhances human keratinocyte migration and proliferation *in vitro* (Engin 1998; Blais et al. 2014), but the effect of the whole sensory nerve secretome has not been characterized. We examined the effect of culture with SN-CM on basal keratinocyte proliferation, migration, differentiation, and metabolism, finding that SN-CM has a varied effect on keratinocytes depending on their state; after scratch injury: slowing migration, increasing basal cytokeratin expression, and increasing mitochondrial activity, under homeostatic conditions: decreasing proliferation, and increasing ATP production. Our results demonstrate that nerve-secreted factors can directly modulate keratinocyte behavior, with outcomes dependent on the cellular state of keratinocytes, which has important implications for the control of re-epithelialization *in vitro* and overall skin wound healing. Although we expected to see an increase in keratinocyte migration and proliferation after scratch based on previous work, which isolated the effect of single factors on keratinocyte function; it is possible that the different outcomes reported are due to our use of the whole DRG secretome and the interactions of multiple factors to produce the resultant change in keratinocyte function. Our results also underscore the complexity of keratinocyte regulation and suggest that nerve-secreted factors may play a variable role in directing keratinocyte function that is dependent on the tissue-level state. Successful wound healing requires precise regulation of keratinocyte functions that are required for each stage of wound healing, homeostatic state, and the location within the layers of the epidermis (Kim and Dao 2023; Bain et al. 2024), as hyper- or hypoactivity at any stage disrupts homeostasis (Wilkinson and Hardman 2020; Zhou et al. 2022). For example, skin disorders such as psoriasis develop due to excessive keratinocyte proliferation, and therapeutics to treat this and other similar skin disorders target a reduction in keratinocyte proliferation and migration (López et al. 2021). The increase in K14 expression post-scratch with SN-CM treatment is in line with the observed decrease in migration and suggests that sensory innervation may contribute to maintaining the basal, non-migratory K14+keratinocyte population rather than promoting the switch to the migratory and proliferative K10+ suprabasal population that is activated following injury (Park et al. 2017; Cockburn et al. 2022). Under basal conditions, the epidermis undergoes complete turnover in 1 month, whereas in pathological conditions such as psoriasis, turnover occurs in 3or 4 days (Halprin 1972). Therefore, based on our results, sensory nerve signaling may contribute to the precise regulation of keratinocyte proliferation, migration, and phenotype to prevent hyperproliferation and prolonged migration. Lastly, there are limitations on extrapolating findings from *in vitro* models to *in situ* wound healing. The effects of SN-CM that we observed on cultured keratinocytes may differ at the tissue level depending on the stage of wound healing investigated and the presence of other important microenvironmental cues found in the skin. Further, *in vivo* research will be required to determine the interaction of other systemic factors with nerve-driven cues and the overall effect on keratinocyte function. Nonetheless, our results provide valuable insight into the role of nerves in directing keratinocyte function and serve as a foundation to further characterize this important relationship during skin wound healing.

### Proteomic profiling of the sensory nerve secretome identified pro-healing and metabolic factors

To identify potential nerve-derived secreted factors involved in cutaneous wound healing, we performed proteomic profiling of SN-CM. Characterization of the sensory nerve secretome is an important step toward understanding the physiological response and pathological mechanisms of wound healing (Engin et al. 1996; Leroux et al. 2020). To our knowledge, this is the first quantitative proteomics study of the mouse lumbar sensory nerve secretome. We isolated lumbar DRG specifically for analysis as previous literature has shown that DRG may exhibit different effects based on spinal region (Carreau et al. 1997; Hordeaux et al. 2020). Using LC-MS/MS analysis, we characterized the secretome profile of the DRG and identified several important proteins related to wound healing such as collagens (collagen type I, type IV), pro-regenerative factors (BMP-1, FGFR-1), proteases (MMP17, MMP2), and ECM proteins (fibrillin-1). We also detected the serine protease serpina3n, which has been previously shown to improve wound healing in diabetic mice by significantly accelerating epidermal migration following a full thickness excision wound (Hsu et al. 2014). While LC-MS/MS is considered a powerful proteomic technique, it adopts a “hypothesis-free” method of protein identification and quantification, such that there are no targets during analysis and a large dataset is generated of specific protein abundance based on what is detected in the conditioned media (Li et al. 2021). Therefore, using these results as a basis, we created a compendium of protein function and processes using the PANTHER classification system to identify pathways and functional groupings of the identified proteins. We identified proteins involved in biological processes such as metabolic processes, catalytic activity, cell motility, and cell division, which is consistent with the effect on keratinocytes that we observed with culture in SN-CM.

Using both LC-MS/MS and a cytokine array we detected an abundance of various MMPs in the DRG secretome that have reported roles in skin wound healing, particularly MMP2 and the inactive form of MMP9, which is consistent with previous literature, as MMP9 and MMP2 are known to be secreted by nerves (Saygili et al. 2010; Lu et al. 2022). We also detected the endogenous inhibitor of MMP2, TIMP2, which plays a critical role in the inflammatory phase of wound healing (Mirastschijski et al. 2004; Patel et al. 2006). The presence of both MMPs and their inhibitors also suggests that nerves may contribute to the temporospatial regulation of MMP activity during wound healing. Previous research has established the importance of MMPs in wound healing, specifically on keratinocyte function, by modulating inflammatory factors (Stevens and Page-McCaw 2012), activating downstream pathways to support healing (Vaalamo et al. 1999), and controlling keratinocyte migration (Mäkelä et al. 1999; Mohan et al. 2002; Stevens and Page-McCaw 2012); therefore it is possible that the identified nerve-derived MMPs contribute toward these important functions. Overall, the secretome database we generated will serve as an important foundation to test identified factors in their role in wound healing, generating new therapeutic targets.

### Keratinocyte metabolism is altered in sensory nerve conditioned media

As the analysis of our proteomic data identified SN-CM contained factors involved in cellular metabolism, we investigated the metabolic profile of keratinocytes cultured in SN-CM. Our findings confirmed that keratinocyte metabolism is not negatively affected by SN-CM and in fact demonstrated that the total amount of ATP produced is significantly increased in keratinocytes cultured with SN-CM when compared to the control group in homeostatic conditions. We also showed that after scratch injury when wound closure is almost complete (72 h), there is an increase in mitochondrial activity in keratinocytes cultured in SN-CM. Altered keratinocyte metabolism has been previously shown to be correlated with dysfunctional wound closure, for example, in epidermal cells of burned skin glycolytic ATP production is increased, and targeting molecules within the glucose metabolic pathway can enhance skin healing following a burn *in vivo* (Hunt and Pai 1972). An increase in ATP production can indicate that cells are undergoing more energetically taxing functions, such as cell division and migration (Li et al. 2019; Rigoulet et al. 2020, Martínez et al. 2020). Although we observed a decrease in these functions when keratinocytes were cultured with SN-CM, there are other cellular mechanisms that require higher energy input. This includes secretion of cytokines, growth factors and proteases that have been shown to be impaired when mitochondrial catabolism is inhibited (Trompette et al. 2022). Additionally, in keratinocytes, mitochondria provide intermediates that support the biosynthetic pathways required for keratin production and lipid metabolism (Hunt and Pai 1972; Salazar-Roa and Malumbres 2017). It is possible that the observed increase in total ATP in keratinocytes cultured with SN-CM may allow cells to undergo other energetically taxing reactions that are involved in normal keratinocyte function. Furthermore, we observed a trend toward more ATP being produced via oxidative phosphorylation in homeostatic keratinocytes cultured with SN-CM, which is consistent with the decrease in keratinocyte proliferation seen with SN-CM culture. Previous literature has shown that hyperproliferative keratinocytes produce ATP anaerobically via glycolysis, which is considered a hallmark of psoriasis and atopic dermatitis (McGill et al. 2005). If glycolytic metabolism is inhibited, keratinocyte proliferation is inhibited both *in vitro* and *in vivo* (McGill et al. 2005). Therefore, in addition to demonstrating that SN-CM did not adversely affect keratinocyte viability and metabolism, our metabolic profiling results also suggest a coupling of the trend toward a shift to oxidative phosphorylation and the observed reduction in keratinocyte proliferation.

Our proteomic classification of secreted factors within SN-CM identified proteins involved in metabolic processes, which correlates with our findings of increased keratinocyte ATP production rate and mitochondrial activity. Specifically, we identified groups of proteins from the nerve secretome that are involved in the citric acid cycle, the pyruvate pathway, glycolysis, as well as ATP synthesis. Using the STRING database to further parse out protein interactions, we found that SN-CM contains proteins involved with ATP production by chemiosmotic coupling, the release of enzyme bound ATP, as well as proteins that stimulate ATPase (e.g., pyruvate kinase, malate dehydrogenase, ATP synthase, hexokinase-1, cytochrome-C, and glucose-6-phosphate isomerase) (Takahashi et al. 2013). In particular, cytochrome-c is involved in the mechanism of action of Anthralin, a drug used to clear psoriatic plaques by reducing keratinocyte hyperproliferation via the activation of caspase-3 pathway, which is consistent with our findings of reduced proliferation despite increased ATP production (Liu et al. 2021). In addition, we detected pyruvate kinase, a key enzyme in glycolysis, which when depleted leads to skin pathologies, as well as disrupted epidermal homeostasis (Liu et al. 2021). Combining this data with our metabolomic analysis of keratinocytes provides evidence that sensory nerves may have a role in regulating keratinocyte metabolism. While our data suggest that sensory nerves can regulate the metabolism of keratinocytes in both homeostatic and post-scratch states, there are some important caveats to our findings. Some of the identified proteins associated with metabolism are also released from damaged cells, which we cannot rule out as possibly occurring during media collection or prolonged DRG culture. Furthermore, while dysfunctional keratinocyte metabolism has been linked to impaired wound healing by others (Lu et al. 2022), it is unclear if these changes in keratinocyte metabolism elicited by sensory nerves translate into changes during wound healing and/or skin homeostasis. Further work is needed to translate our findings into an *in vivo* system to assess the impact of nerve-mediated changes in keratinocyte function on tissue-level dynamics.

## Conclusion

Non-healing wounds are the result of dysfunctional re-epithelialization causing a risk of infection, pain, and reducing quality of life (Usui et al. 2008; Colenci and Patricia 2018; Wang et al. 2023). While peripheral innervation is important for wound healing, data are lacking for the role of sensory nerves during re-epithelialization, and specifically for keratinocyte function. We provide *in vitro* evidence that sensory nerves secrete trophic factors that control the migration, proliferation, and metabolism of keratinocytes—functions whose regulation is essential for skin homeostasis, re-epithelialization, and overall wound healing (Smith and Liu 2002). Using proteomic analyses of sensory nerve conditioned media, we characterized the secretome profile of sensory nerves and identified key trophic factors that mediate wound healing, including through modulation of keratinocyte metabolism. This work provides foundational knowledge of the potential mechanisms controlling keratinocyte function to increase our understanding of the role of peripheral nerves in cutaneous wound healing. Our findings can also extend to other cell types of the skin that are important for wound healing, such as dermal fibroblasts, to provide a basis to study the role of the identified peripheral nerve-derived factors on the function of these cell types. Understanding the complex cellular interactions that drive wound healing will help to target the development of therapeutic strategies to treat non-healing wounds.

## Supplementary Material

obaf009_Supplemental_Files

## Data Availability

The mass spectrometry proteomics data have been deposited to the ProteomeXchange Consortium via the PRIDE [1] partner repository with the dataset identifier PXD062182.
