# Supplementary material for: Sensory nerve-secreted factors regulate basal keratinocyte function in vitro
Source: Integr Org Biol. 2025 Mar 3;7(1):obaf009. doi: 10.1093/iob/obaf009 (PMC11945292; doi:10.1093/iob/obaf009)
Supplement: obaf009_Supplemental_Files [file obaf009_supplemental_files.zip › Supplementary Data.docx]

**Supplementary Data**

**Supplemental Figure 1: Keratinocyte viability is maintained in sensory nerve-conditioned media.** PrestoBlue cell viability assay examining the effects of sensory nerve conditioned media on keratinocyte viability after 0, 1 and 7 days in culture**.** Experimental groups include keratinocytes cultured in base keratinocyte media (10% FBS, DMEM/F12), keratinocytes cultured in a 1:1 ratio of keratinocyte media and unconditioned sensory nerve media (UM; complete neurobasal), and keratinocytes cultured in a 1:1 ratio of keratinocyte media and sensory nerve conditioned media (CM). There were no significant differences after one day of culture in all conditions. There was a significant increase in keratinocyte viability at 7 days when cultured in sensory nerve media (**p = 0.0139, n = 3 biological replicates). These data indicate that culture in SN-CM does not negatively impact keratinocyte viability. Two-way ANOVA analysis was used at each timepoint between groups. Data expressed as mean ± SD.

**Supplemental Figure 2: Low levels of proliferation detected at the scratch site in keratinocytes exposed to sensory nerve conditioned media (SN-CM) in serum reduced conditions.** Keratinocytes cultured in SN-CM and control medium were fixed at 48 and 72 hours in 4% PFA and stained with Ki67 to assess proliferation and the wound site (*n* = 3 biological replicates). No significant differences in Ki67 positive nuclei were observed between SN-CM and the control. Unpaired t-tests were performed between each group at each timepoint. Data expressed as mean ± SD.

**Supplemental Figure 3: All proteins detected in sensory nerve conditioned media and unconditioned media using LC-MS/MS.** Hierarchically clustered heat map of the log_2_ protein quantity of three biological replicates of SN-CM and the blank media control. Columns represent sample replicates and rows represent detected proteins. Cells are coloured based on overall protein abundance, yellow represents the most highly detected within that sample and dark blue the lowest detected. Blank spaces represent the absence of the identified protein within that sample.
