# Supplementary figures and images for: Sensory nerve-secreted factors regulate basal keratinocyte function in vitro
Source: Integr Org Biol. 2025 Mar 3;7(1):obaf009. doi: 10.1093/iob/obaf009 (PMC11945292; doi:10.1093/iob/obaf009)

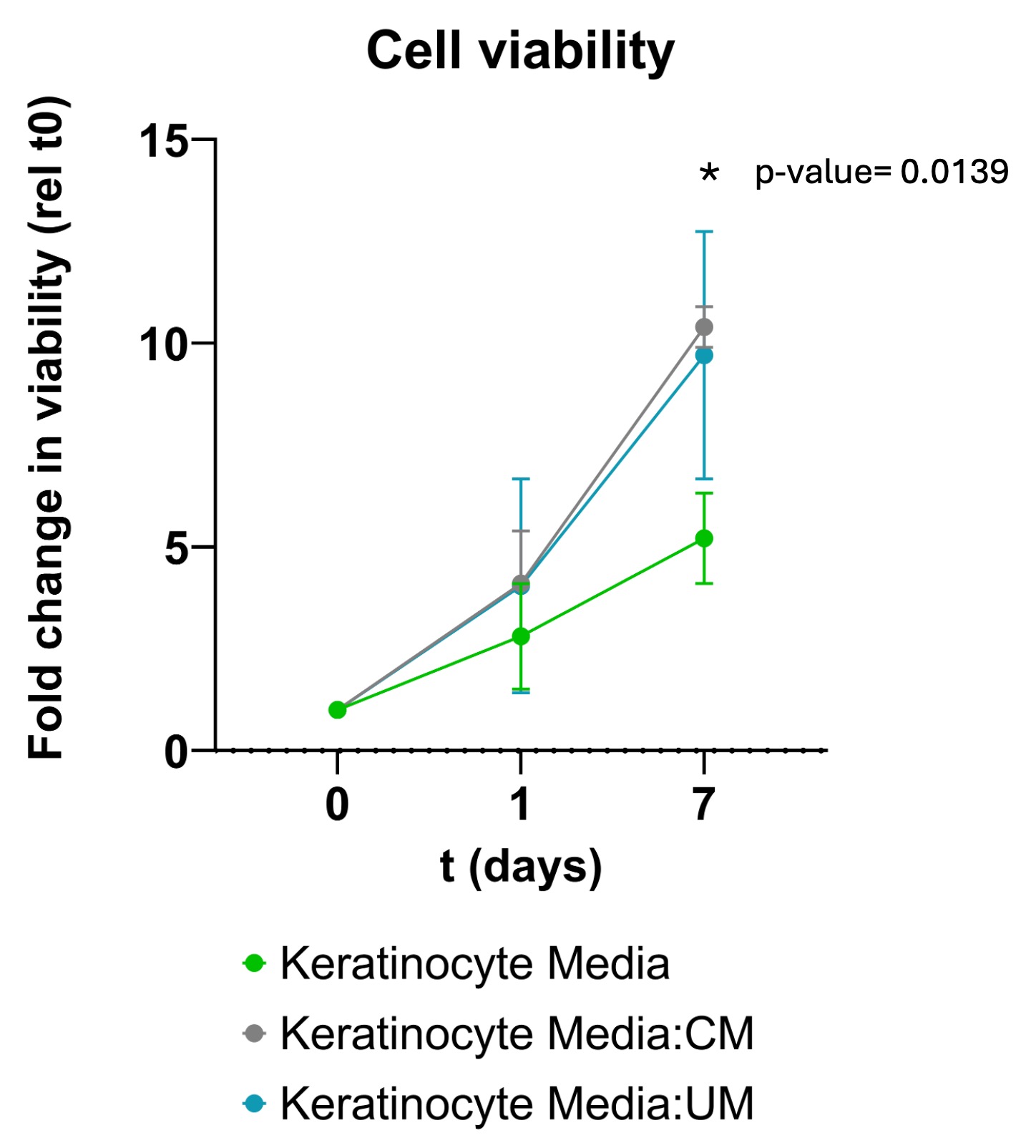

Supplement: obaf009_Supplemental_Files [file obaf009_supplemental_files.zip › Supplemental Figure 1.jpg]

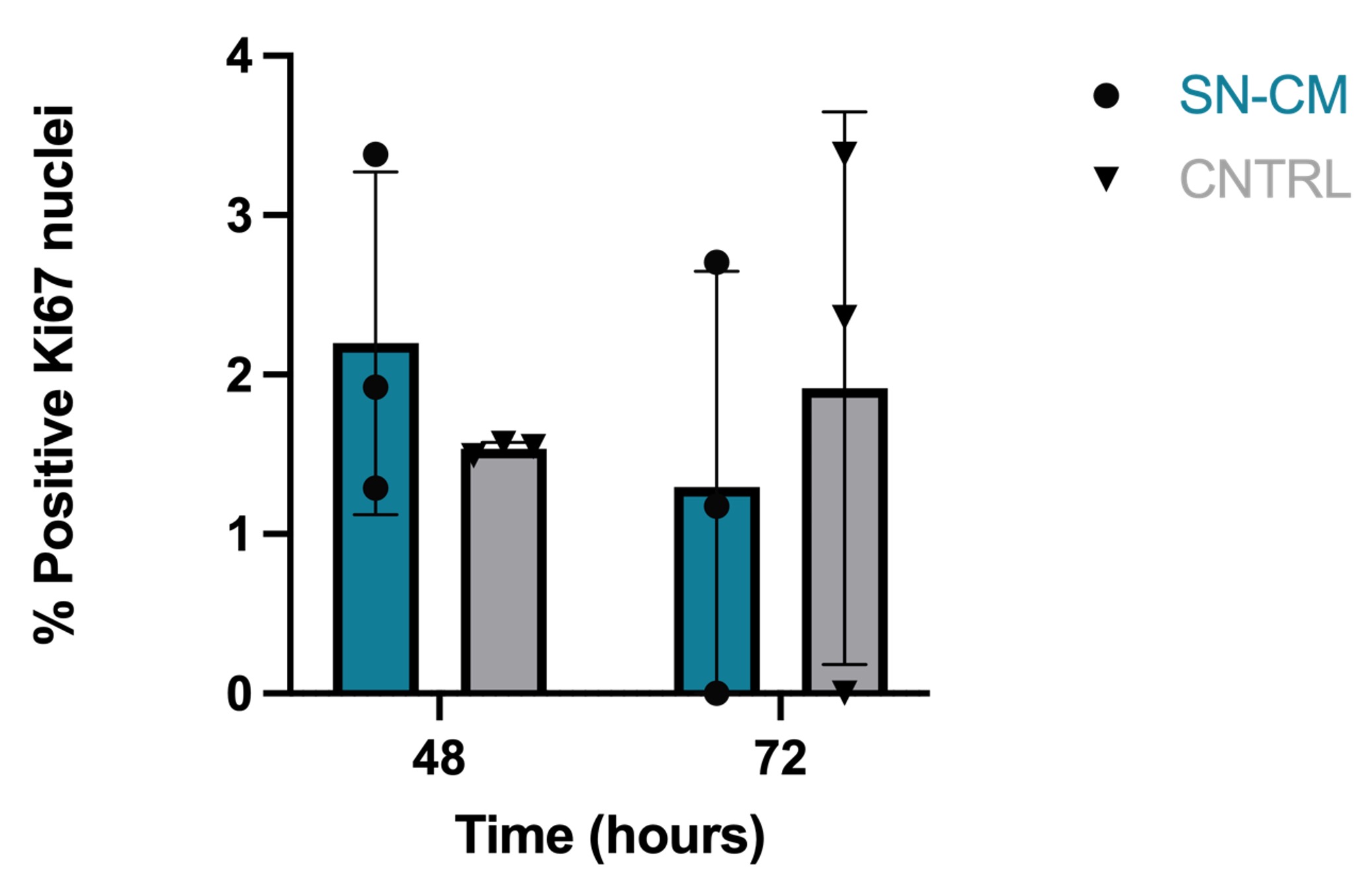

Supplement: obaf009_Supplemental_Files [file obaf009_supplemental_files.zip › Supplemental Figure 2.jpg]

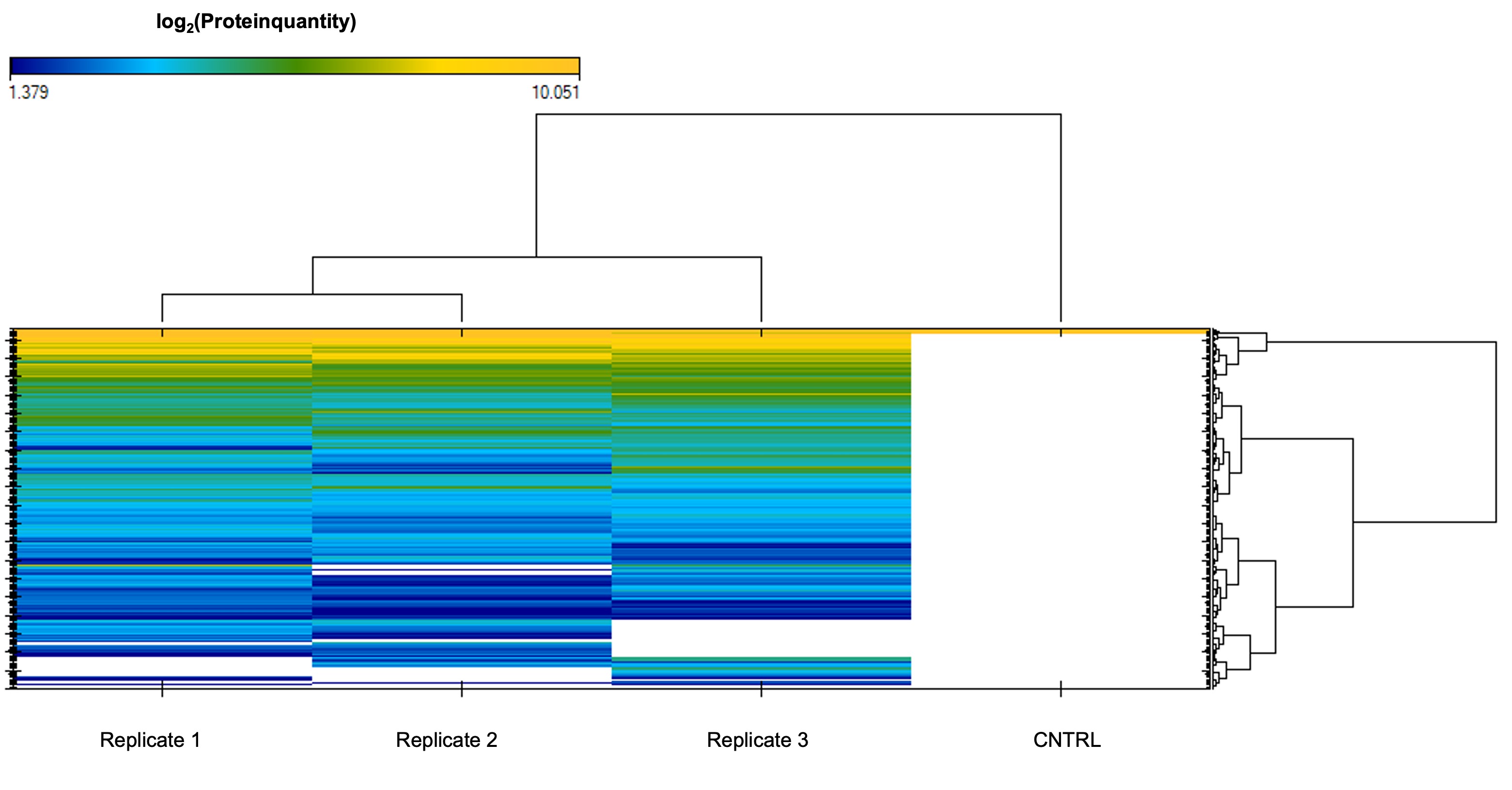

Supplement: obaf009_Supplemental_Files [file obaf009_supplemental_files.zip › Supplemental Figure 3.jpg]
